# Year-Round Tracking of Small Trans-Saharan Migrants Using Light-Level Geolocators

**DOI:** 10.1371/journal.pone.0009566

**Published:** 2010-03-05

**Authors:** Erich Bächler, Steffen Hahn, Michael Schaub, Raphaël Arlettaz, Lukas Jenni, James W. Fox, Vsevolod Afanasyev, Felix Liechti

**Affiliations:** 1 Swiss Ornithological Institute, Sempach, Switzerland; 2 Institute of Ecology and Evolution, Conservation Biology, University of Bern, Bern, Switzerland; 3 British Antarctic Survey, Natural Environment Research Council, Cambridge, United Kingdom; University of Hull, United Kingdom

## Abstract

Since 1899 ringing (or banding) remained the most important source of information about migration routes, stopover sites and wintering grounds for birds that are too small to carry satellite-based tracking systems. Despite the large quantity of migrating birds ringed in their breeding areas in Europe, the number of ring recoveries from sub-Saharan Africa is very low and therefore the whereabouts of most small bird species outside the breeding season remain a mystery. With new miniaturized light-level geolocators it is now possible to look beyond the limits of ring recovery data. Here we show for the first time year round tracks of a near passerine trans-Saharan migrant, the European Hoopoe (*Upupa epops epops*). Three birds wintered in the Sahel zone of Western Africa where they remained stationary for most of the time. One bird chose a south-easterly route following the Italian peninsula. Birds from the same breeding population used different migration routes and wintering sites, suggesting a low level of migratory connectivity between breeding and wintering areas. Our tracking of a near passerine bird, the European Hoopoe, with light-level geolocators opens a new chapter in the research of Palaearctic-African bird migration as this new tool revolutionizes our ability to discover migration routes, stopover sites and wintering grounds of small birds.

## Introduction

Up to present, the difficulties in tracking small animals, particularly birds, throughout an entire migratory cycle have impeded our understanding of migratory connectivity [1]. Indirect methods such as stable isotopes [2] or genetic markers [3] have provided useful insights, but for tracking animal movements, these techniques still have important drawbacks and provide information on large geographical scales only [1], [4]. Amongst the direct tracking methods, satellite telemetry has been the most important advance in recent years. However, the current size and weight of satellite tags is too large for equipping small birds [1], which represent the vast majority of migratory birds [5]. Due to these limitations ringing (or banding), introduced in 1899 [6], has remained the most important source of our knowledge about large scale movements of small birds [7]. However, ringing has many limitations itself [6], especially the usually very low and spatially highly heterogeneous recovery rate [7]. This is particularly true for Africa, where reports of recoveries are rare due to the low population density and because people are less aware of the significance of such recoveries [8], [9].

The rapid advance in miniaturization of electronic devices has led to the development of a new powerful tool for studying migratory movements of small birds: light-level geolocators [10], [11]. These data loggers measure levels of sunlight and store them in an internal memory together with a time stamp. Birds are equipped with geolocators in the breeding area and have to be recaptured for data download after returning from their wintering grounds. Once recovered, sunrise and sunset times can be determined from the recorded light level data for the elapsed time period. Latitude and longitude can then be calculated based on day length and local apparent noon and midnight, respectively [12]. The accuracy of longitude determination is equal throughout the year, whereas determination of latitude is most precise at solstices and impossible around equinoxes. Furthermore, position estimates can be affected by shading, clouds, latitude and the animals movement and behaviour [12]. A comparison between satellite transmitters and geolocators on albatrosses indicated a geolocator location accuracy relative to satellite transmitter fixes of 186 km (s.d.: ±114 km) [13]. This error may be somewhat larger for land birds as they can enter dense vegetation or cavities and because topography can shorten apparent day length.

We used light-level geolocators to track European Hoopoes (*Upupa epops epops*) breeding in the Valais, an inner-Alpine valley in Switzerland, to their wintering grounds and back. The aims of the study were to test the suitability of the technique for Hoopoes, to assess whether the recapture rate of Hoopoes is influenced by geolocators and to gain first insights into migration routes and wintering sites of Hoopoes. European Hoopoes are supposed to winter in the Sahel zone from the Atlantic coast to the Horn of Africa, including the Great Rift Valley [14]. Of all Hoopoes ringed between 1914 and 2005 all over Europe, only four ringed birds have been recovered in Africa, three at the coast of northern Africa and only one individual in the presumed sub-Saharan wintering area at the southern fringe of the Sahara desert [15]. In our study population 4101 birds have been ringed between 1998 and 2008 but none of these rings were recovered in Africa (own unpublished data).

## Materials and Methods

### Ethics Statement

All animals were handled in strict accordance with good animal practice as defined by the Swiss Federal office for the environment (animal protection guideline 4.03). All animal work was approved by the Swiss Federal office for the environment and the nature protection agency of Canton of Valais.

### Study Site and Capture

The study area is situated in the plain of an inner-Alpine valley and spreads over a surface of about 62 km^2^. The plain is used intensively for agricultural purposes, mainly fruit tree plantations and vineyards. With very few exceptions, Hoopoes are breeding in artificial nest boxes, placed in small sheds used for agriculture. A total of 625 nest boxes was available in 2008. During the breeding season 2008 we equipped 19 adult breeding Hoopoes (11 males and 8 females) with geolocators. Capture in 2008 and recapture in 2009 were carried out within the framework of a project for studying the population dynamics of Hoopoes, where all nest boxes were controlled every two weeks during the breeding season. Nest boxes occupied by Hoopoes were controlled every three days and the birds were captured and ringed. As all nest boxes were controlled regularly, no additional effort was made to find birds carrying geolocators. Recapture rates should therefore not be biased towards birds carrying geolocators.

### Geolocators

We used Mk14S geolocators developed by British Antarctic Survey (BAS) and attached them to Hoopoes using leg-loop harnesses [16] consisting of silicone. Devices weighed 1.8 g including harness. Birds with geolocators that were recaptured in 2009 had a median weight of 70.5 g (min – max  = 63 g–82.5 g) at the time they were equipped with geolocators. Thus the geolocator weight corresponds to a median percentage of 2.6% (min - max  = 2.2%−2.9%) of the birds body weight. Birds with geolocators that were not recaptured in 2009 had a mean weight of 69 g (min – max  = 62.7 g–82 g) at the time they were equipped with geolocators. The light sensor was mounted on a stalk of 2 cm in length to prevent it from being covered by the feathers of the birds. Geolocators measured light-levels every 60 seconds and stored the maximum value within a 10 min interval.

Light-level data were linearly corrected for clock drift using program Bastrak (BAS) and visually checked for shading events. Only continuously rising or falling light curves with a duration of 20 to 30 minutes between maximum and minimum light-level were used. Latitude was not calculated for 15 days before and after vernal and autumnal equinox. Based on a pre-deployment calibration at a known location without shading we determined light-level thresholds corresponding to a solar elevation angle of –6°. These thresholds were then used to determine sunrise and sunset times using program TransEdit (BAS). Apparent day length at the breeding areas of the Hoopoes (an inner-Alpine valley) was shortened due to surrounding mountains up to 3000 m. a. s. l. and therefore a calibration of the geolocators on the birds was not possible. Positions were calculated with software BirdTracker (BAS). Both, midnight and noon locations were used and BirdTracker's movement compensation option was applied as it is unclear whether Hoopoes are day- or night-migrants.

### Data Analysis

Wintering areas were derived from latitude and longitude data. Important stopover sites were identified by applying a circle with a radius of 200 km around each point outside the wintering areas. If a circle encompassed more than five points (corresponding to a time span of 48 hours), these points were assigned to stopover sites. Kernel density estimation (program ArcMap 9.2, ESRI) was then applied separately for wintering areas, stopover sites on autumn migration and stopover sites on spring migration on a UTM31N projection. Search radius was set to 200 km and a grid size of 2 km was used. We calculated kernel densities encompassing 50%, 75% and 90% of the maximum density. The arrival and departure dates for the stopover sites and wintering areas were then determined from the positioning data. Where latitude data was not available due to equinoxes or shading events, departure and arrival dates were estimated from longitude data only. The course of the migration routes between stopover sites and wintering areas was derived directly from the positioning data.

Because it was not always possible to determine arrival and departure times at wintering grounds due to equinoxes, we used longitude data to estimate overall migration rates. We defined the minimum duration of migration as the time the bird spent between the longitude of the breeding site and that of the wintering area. Overall migration distance was defined as the minimum distance between breeding, stopover and wintering sites. Overall migration rate was then calculated as migration distance/minimum duration of migration.

## Results

Five Hoopoes carrying geolocators (26.3%) were recaptured in the study area in 2009 (3 females and 2 males). From the 111 birds ringed in 2008, 25 individuals (22.5%) were recaptured in 2009. There was no statistical difference in the recapture rate between ringed birds and those carrying rings and geolocators (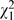 = 0.08, P = 0.77, n = 160). Because nearly all Hoopoes in our study area are breeding in artificial nest boxes, we assume that we were able to catch most of the birds present in the area. Therefore the relatively low recapture rate is better explained by dispersal and mortality.

**Figure 1:**
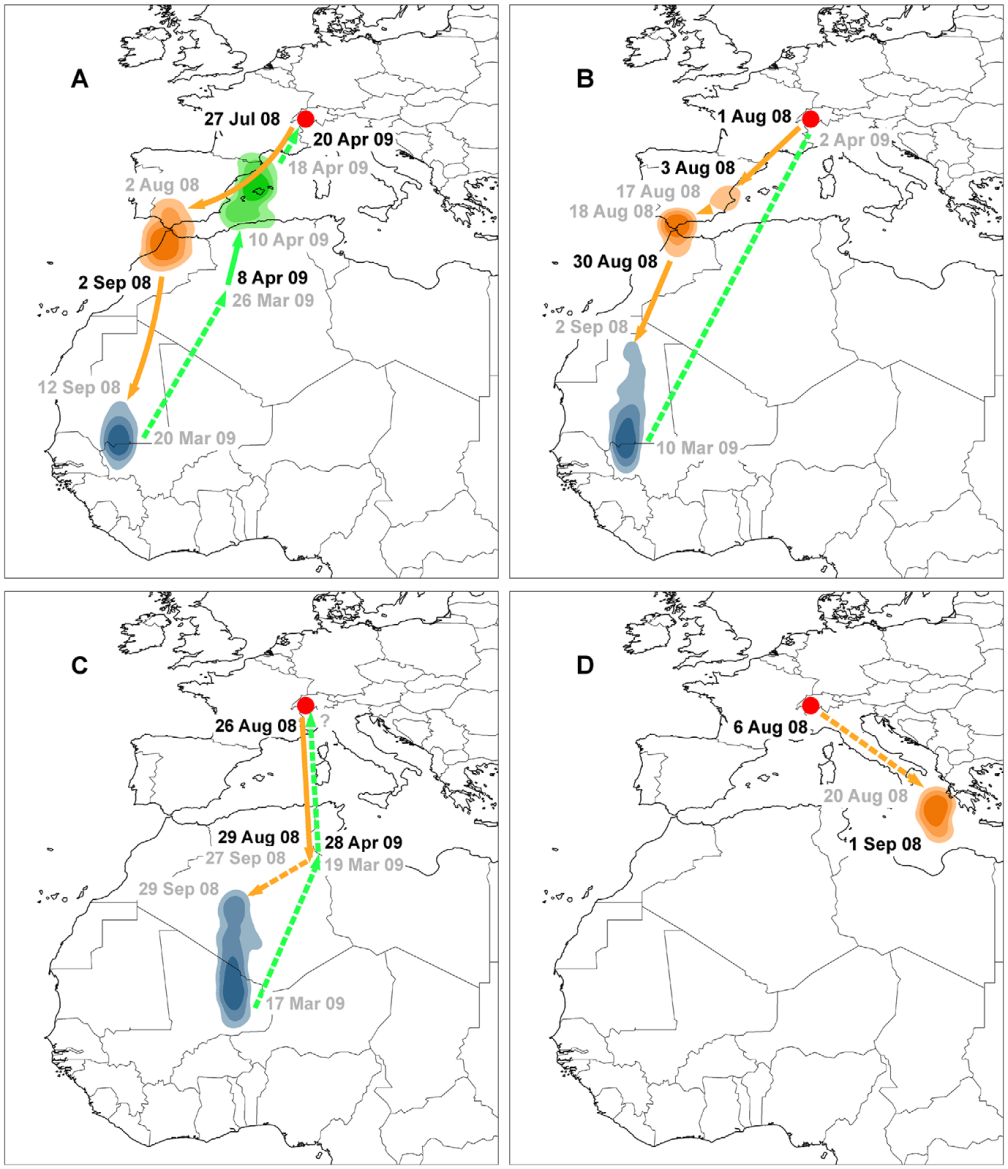
Migration routes, staging- and wintering areas of Hoopoes. Breeding site (red point), staging areas and wintering grounds (density contours) of two female (A,B) and two male (C,D) hoopoes with estimated arrival and departure dates. Density contours reflect 50%, 75% and 90% kernel density. Autumn migration routes and stopover sites are colored orange, spring migration routes and stopover sites green and wintering sites blue. Arrival and departure dates in black are derived from longitude and latitude data, arrival and departure dates in grey are estimated from longitude data only. Solid arrows indicate migration routes derived directly from positioning data, dashed arrows stand for unknown migration routes. Uncertainties in migration routes and dates are due to equinoxes and shading events in the light-level data.

One of the recaptured birds had lost the geolocator en route. The remaining birds originating from the same small breeding population showed three different autumn migration routes (Figure 1). While both females followed a western flyway via the Strait of Gibraltar, male C crossed the Mediterranean Sea along an axis situated between the Balearic islands and Corsica and Sardinia, while male D chose an eastern flyway following the Italian peninsula. After a stay of approximately one week at the south-eastern coast of Italy this bird was located in the Ionian Sea. As there are no islands around this position the most probable location lies at the Albanian-Greek coast of the Ionian Sea. The southern shift of the position data can be explained by the influence of the mountainous region on apparent day length. The two females wintered in the same region close to the border of Mauritania and Western Mali where they remained stationary during about half a year. Male C wintered about 1,000 km farther east, first in central Algeria, then mainly in eastern Mali. Unfortunately the geolocator of male D stopped collecting data on 1 September 2008 and hence it was not possible to determine its wintering ground and spring migration route. Female A returned back to the breeding ground by following a loop migration pattern via Adrar Province (Algeria) and the Balearic islands. Female B migrated around vernal equinox, when it is impossible to calculate latitude. On its way back to Europe male C stopped over in the border region of Algeria and Tunisia for at least two weeks. The further course of its spring migration could not be determined precisely due to many shading events in the light-level data.

Based on the longitudinal data, which are not affected by equinoxes, we estimated overall spring and autumn migration rates for the two females (see methods). Female A spent 48 days on migration in autumn and 32 days during spring. Mean daily speed during spring migration was 122 km d^−1^ and thus 1.5 times faster than in autumn where the bird covered 81 km d^−1^ on average. Female B needed 33 days to reach the wintering area and 24 days to return to the breeding ground. Mean daily speed of this bird was 118 km d^−1^ in autumn. In spring it migrated 1.4 times faster, covering 163 km d^−1^ on average. These findings are in line with the results from earlier studies [17], [18] supporting the idea that time constraints for Palaearctic migrants differ between autumn and spring migration [19].

## Discussion

The results from this study demonstrate the enormous potential of light-level geolocators for studying migration of small birds. With the application of only 19 geolocators (4 recovered), we gained by far more information about Hoopoe migration than with the 4101 birds ringed in this population during the last 10 years and much more information about wintering areas than from all Hoopoes ever ringed in Europe. We found the first direct evidence of loop migration from an individual near passerine trans-Saharan migrant and different migration routes and wintering sites within a single population. Based on ring recovery data, Reichlin et al. [15] proposed a migratory divide for European hoopoes running through central Europe in close proximity to our study site. This could be one possible explanation for the low migratory connectivity between breeding and wintering areas we found in our study population. Birds breeding in eastern and western Europe may possibly show a higher migratory connectivity.

New miniaturized geolocators allow for unraveling migratory routes, stopover sites and wintering areas of small birds in space and time. The knowledge about the seasonal whereabouts of small migratory birds opens new and fascinating perspectives in ecological research and conservation alike. For example, tracking individuals during the complete year allows to study the influence of the encountered environmental conditions on individual reproductive decisions and success. Predictions of the effect of global climate change on populations of migratory birds will become much more accurate, because they can be based on weather data that are spatially and temporally explicit. Routes of pathogens transmitted by migratory birds can be identified and finally, such data will allow to evaluate whether current declines of migrant populations in the northern hemisphere [20] are due to deteriorations of environmental conditions at stopover sites and wintering areas, which in turn will guide more efficient conservation actions.
